# Case report: BTK inhibitors is effective in type II mixed cryoglobulinemia with wild-type MyD88

**DOI:** 10.3389/fimmu.2024.1390958

**Published:** 2024-05-03

**Authors:** Mingyue Xu, Yunyu Xu, Li Yuan, Da Shang, Ruiying Chen, Shaojun Liu, Yan Li, Aiping Liu, Ruilai Liu, Qian Wang, Tianling Ding, Qionghong Xie, Chuan-Ming Hao

**Affiliations:** ^1^ Division of Nephrology, Huashan Hospital, Fudan University, Shanghai, China; ^2^ Division of Nephrology, The Second Affiliated Hospital of Guangxi Medical University, Nanning, China; ^3^ Clinical Laboratory of Huashan Hospital, Fudan University, Shanghai, China; ^4^ Division of Hematology, Huashan Hospital, Fudan University, Shanghai, China

**Keywords:** type II cryoglobulinemia, BTK inhibitors, MyD88 mutation, hepatitis B virus, ibrutinib, orelabrutinib

## Abstract

This study presents two cases of type II mixed cryoglobulinemia. One case is essential, while the other is presumably associated with hepatitis B virus (HBV) infection. Both patients tested positive for monoclonal IgMκ, but negative for MyD88 mutation. They showed resistance to rituximab combined with a glucosteroid regimen, but responded positively to BTK inhibitors. These cases highlight the remarkable effectiveness of BTK inhibitors in treating refractory type II cryoglobulinemia without MyD88 mutation. The first patient achieved rapid complete remission of nephrotic syndrome within one month of starting ibrutinib, along with a significant reduction in cryoglobulin levels and abnormal clonal cells. The second patient had a rapid disappearance of rash within three days and accelerated wound healing within one week of initiating orelabrutinib, accompanied by a reduction in C-reactive protein. However, there was no reduction in cryoglobulin levels during the 12-month follow-up. These findings suggest varied mechanisms of action of BTK inhibitors in type II cryoglobulinemia through different mechanisms.

## Introduction

Cryoglobulinemia is characterized by the presence of immunoglobulins in the bloodstream that precipitate at lower temperatures and dissolve again when warmed. Brouet et al (1) classified cryoglobulinemia into three types based on immunoglobulins composition. Type II cryoglobulinemia comprises a mixture of monoclonal IgM with rheumatoid factor (RF) activity and polyclonal IgG. Approximately 60% to 95% of type II cryoglobulinemia cases are secondary to chronic hepatitis C virus (HCV) (2), while others may be secondary to chronic hepatitis B virus (HBV), autoimmune diseases, and lymphoproliferative disorders. Cases without identifiable causes are considered essential mixed cryoglobulinemia. Patients with symptomatic cryoglobulinemia may exhibit various manifestations of vasculitis, including purpura, leg ulcers, membranoproliferative glomerulonephritis (MPGN), and peripheral neuropathy. Treatment depends on the extent of organ damage and the presence of underlying diseases, typically targeting the underlying disorder or suppressing the immune system.

Bruton’s tyrosine kinase (BTK) is a crucial enzyme involved in B-cell activation and proliferation. Approximately 93–97% of patients with Waldenstrom macroglobulinemia have a somatic mutation in MyD88, which triggers tumor growth by activating NF-κB signaling via BTK (3). BTK inhibitors are well recognized for their effectiveness in treating Waldenstrom macroglobulinemia with MyD88 mutation (4). Besides B cells, BTK exerts influence on T cells, macrophages, neutrophils, NK cells, and monocytes via various pathways (5). Consequently, BTK inhibitors have been tested in inflammatory diseases, autoimmune diseases, and infections due to their anti-inflammatory properties (6). Here, we successfully treated two patients suffering from refractory type II mixed cryoglobulinemia without MyD88 mutation with BTK inhibitors.

## Case 1

**Figure 1:**
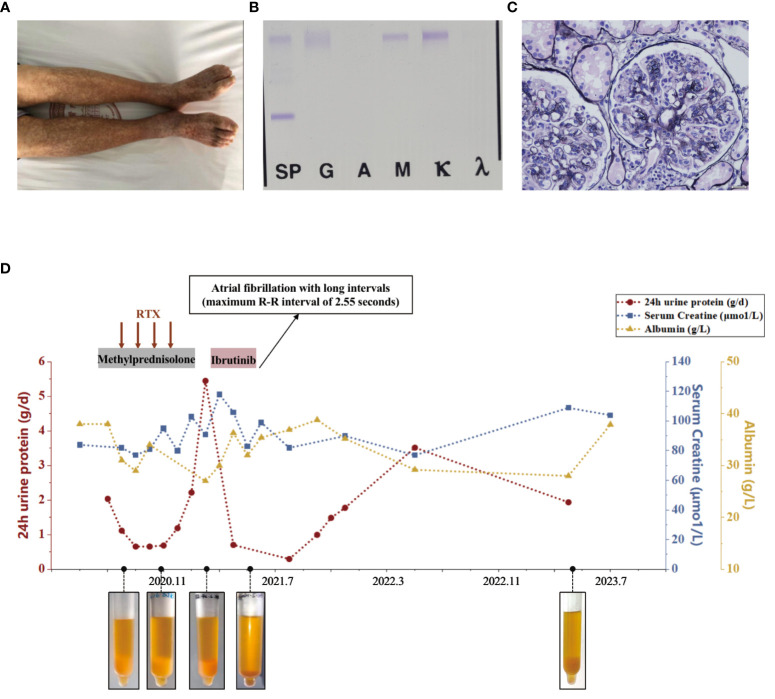
Diagnostic evaluation and clinical course of Case 1. **(A)** Scattered light red rashes on both lower extremities. **(B)** Immunofixation electrophoresis. A monoclonal IgM κ and polyclonal IgG. **(C)** Light microscopy. Membranoproliferative glomerulonephritis pattern. **(D)** Clinical course in case 1 with associated pertinent laboratory values from time of diagnosis to last follow-up.

In July 2020, a 77-year-old male was referred to our department due to persistent proteinuria for the past 6 months. Two years prior to his presentation, he had experienced scattered light red rashes on both lower extremities, which did not protrude from the skin surface or fade under pressure, accompanied by numbness. His hemoglobin level, liver function, and renal function were normal, and he denied any history of diabetes. During subsequent follow-up, he reported worsening bilateral numbness in the lower extremities, along with weakness and tingling sensation. Approximately a year ago, he received methylprednisolone due to peripheral neuropathy, but experienced no improvement. His rash extended to involve the knee joints (as shown in **Figure 1A**), with increased tingling sensation, leading to unstable walking. Additionally, he gradually developed bilateral pitting edema in the lower extremities along with 3+ proteinuria.

Upon presentation to our department, laboratory tests revealed a urine protein level of 2.2g/day, serum creatinine of 82μmol/L, albumin of 38g/L, and hemoglobin of 93g/L. C-reactive protein levels were within normal range. Serology showed positive RF activity (exceeding the upper limit) and negative ANA, anti-dsDNA, ENA as well as ANCA. Complement testing indicated C4 levels <0.068g/L and C3 levels of 0.530g/L. HIV, HBV, and HCV tests returned negative results. Cryoglobulin testing was positive, and immunofixation of cryoglobulin revealed a monoclonal IgM κ and polyclonal IgG, characterized of type II cryoglobulinemia (as shown in **Figure 1B**). Skin biopsy suggested leukocytoclastic vasculitis. Nerve biopsy showed chronic axonal damage with massive perivascular infiltration of numerous mononuclear cells. Renal biopsy revealed MPGN with suspected cryoglobulinemic renal damage (as shown in **Figure 1C**). Immunofluorescence showed IgG (++), IgM (2+~3+), IgA (-), C3 (+), C1q (-), κ (+), and λ (+/-). Bone marrow biopsy indicated abnormal lymphocytes accounting for approximately 2%, and flow cytometry showed abnormal small and medium B lymphocytes (CD19+, CD20+, CD22+, CD5-, CD10-, slgM+, cyκ+, cyλ-) occupying 3% of the nucleated cells, and abnormal plasma cells (CD38+, CD138+part, CD27+, CD19+, CD56-, CD28-, CD117-, restricted expression of cyκ+) occupying 0.37% of nucleated cells. No pathogenic mutations associated with Waldenström macroglobulinemia were found in the MYD88 genes. Consequently, he was diagnosed with type II mixed cryoglobulinemic vasculitis, MPGN, peripheral neuropathy, and purpura of the lower limbs.

The patient received rituximab treatment at a dose of 375mg/m^2^ per month continuously for 4 months, combined with steroids (starting at 30mg/day and tapering off). Subsequently, he had a clinical improvement with a decrease in proteinuria to 0.66g/day. However, his cryoglobulin levels did not decrease significantly. Four months after the last dose of rituximab (March 2021), he developed nephrotic syndrome with proteinuria increasing to 5.45g/day and serum albumin of 27 g/L, although his serum creatinine remained stable. Then he received ibrutinib (420mg/day) treatment. One month later, his nephrotic syndrome achieved complete remission with urine protein of 0.3g/day and albumin of 35.4g/L. His cryoglobulin almost disappeared and his hemoglobin increased to 115g/L, while RF remained at a high level (exceeding the upper limit) and C4 at a low level (<0.068g/L). However, he suffered atrial fibrillation with long intervals (maximum R-R interval of 2.55 seconds) which led to dizziness. Therefore, he discontinued the treatment of ibrutinib. One year later, his 24-hour UP increased to 3.523g/day. His most recent follow-up (July 2023) revealed hemoglobin of 102g/L, albumin of 28 g/L, serum creatinine of 109μmol/L, C3 of 0.480g/L, and C4 of <0.068g/L. His cryoglobulin increased again, in line with the first evaluation. Bone marrow biopsy revealed abnormal lymphocytes accounting for approximately 4.07%, and flow cytometry showed abnormal small and medium B lymphocytes occupying 1.11% of the nucleated cells, and abnormal plasma cells occupying 0.10% of nucleated cells. We have summarized the clinical course in **Figure 1D**.

## Case 2

**Figure 2:**
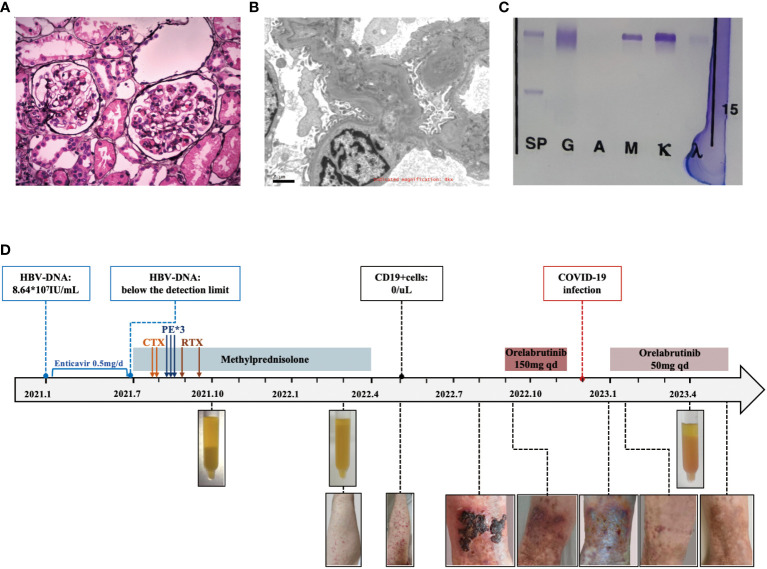
Diagnostic evaluation and clinical course of Case 2. **(A)** Light microscopy. Membranoproliferative glomerulonephritis pattern. **(B)** Electron microscopy. electron-dense deposits in the mesangial area. **(C)** Immunofixation electrophoresis. A monoclonal IgM κ and polyclonal IgG. **(D)** Clinical course in case 2 with associated skin manifestations from time of diagnosis to last follow-up.

A 51-year-old female was admitted in October 2021 due to recurrent rashes of varying sizes with residual pigmentation on both lower extremities for one year. She had previously received low-dose steroids, but saw no improvement. Subsequently, her rash spread to the limbs and back, accompanied by knee joint pain and numbness in the dorsal foot after physical exertion. In January 2021, she tested positive for hepatitis B virus surface antigen (HBsAg) with HBV-DNA of 8.64*10^7^IU/mL, and then she received entecavir treatment. In July 2021, laboratory evaluations showed RF positivity with a significantly decreased complement C4. The blood free light chain κ/λ ratio was 5.675 (elevated), and cryoglobulin was positive. Bone marrow flow cytometry revealed abnormal plasma cells (CD38+, CD19+, CD138+, cκ+, CD20-, CD56-, CD117-, cλ-) accounting for approximately 0.25% of the nucleated cells. Her HBV-DNA levels decreased below the detection limit. Her ANA, dsDNA, and ANCA were negative. The urine protein was 0.1g/day and serum creatinine was 61μmol/L. The cryoglobulin tested positive, and immunofixation of the cryoglobulin revealed a monoclonal IgM κ and polyclonal IgG, consistent with type II mixed cryoglobulinemic vasculitis (as shown in **Figure 2C**). Skin biopsy suggested leukocytoclastic vasculitis. Renal biopsy revealed MPGN with hemosiderin deposits in the mesangial area and thrombus-like material deposition in a few glomerular capillary lumens, consistent with cryoglobulin-related renal damage (as shown in **Figure 2A**). Immunofluorescence showed IgG (+/-), C3 (2+), IgM (1+), κ (2+), and λ (+/-) in the mesangial area and capillary wall, while Fib, IgA, C1q, and Alb were negative. Electron microscopy showed electron-dense deposits in the mesangial area (as shown in **Figure 2B**). She then received entecavir, along with a steroid combined with intravenous cyclophosphamide regimen. However, her purpura reappeared as the glucocorticoid was tapered. Subsequently, she underwent three sessions of plasmapheresis, followed by 2 doses of rituximab (1g per infusion), after which her rash almost disappeared. One month later, her methylprednisolone was reduced to 20mg/day and was subsequently tapered.

In May 2022, She presented with recurrent widespread non-blanching purpura, anemia, inflammation, and persistently high levels of RF and low C4. Her cryoglobulin levels significantly increased, while CD19^+^ cells remained undetectable. No pathogenic mutations related to Waldenstrom macroglobulinemia in the MyD88 and CXCR4 genes. As there was no life-threatening organ damage, she continued antiviral treatment and methylprednisolone (10mg qd).

In September 2022, she developed ulceration on her lower limb and experienced difficulty walking due to pain. She then started orelabrutinib treatment (150mg qd), and her fresh rash completely disappeared two days later. A week later, her ulcerated wound healed, and she was able to walk normally again. She continued taking orelabrutinib for 3 months but discontinued in December 2022 due to a COVID-19 infection. One week after discontinuation, her rash recurred and she also exhibited signs of fatigue and arthralgia. She then resumed orelabrutinib three weeks later at a lower dosage (50mg qd), and the symptoms improved within 3 days. In April 2023, her hemoglobin was 92g/L, CRP was 9.17, and urine protein was 0.33g/day with stable serum creatinine but significantly low level of C3 (0.304g/L) and C4 (<0.066g/L). Her serum cryoglobulin remained at a high level, including IgG at 2.36g/L and IgM at 2.84g/L. The levels of HBsAg, HBcAb, RF-IgG, and RF-IgM were higher in the cryoglobulins than those in the cold supernatant. (Detailed in **Table 1**) The bone marrow flow cytometry showed abnormal B lymphocytes accounting for approximately 0.27%, and abnormal plasma cells for 0.82% of the nucleated cells, suggesting no improvement in abnormal clonal cells. We have summarized the clinical course in **Figure 2D**.

**Table 1:** Composition of various components in cryoglobulin precipitation and serum.

	Cryoglobulin precipitation	Cold supernatant
HBsAg (IU/ml)	6813.58	2910
HBsAb (IU/ml)	0	<2
HBeAg (IU/ml)	0.38	0.13
HBeAb (IU/ml)	1.2	0.86
HBcAb (IU/ml)	5.7	0.01
RF-IgG (U/ml)	123.9	8.1
RF-IgA (U/ml)	16.7	<3
RF-IgM (U/ml)	>300	17.8

## Discussion

We report on two cases of type II cryoglobulinemia. One is essential, while the other is likely associated with HBV infection. Both tested positive for monoclonal IgMκ, but negative for the MyD88 mutation. They initially showed poor response to rituximab combined with glucosteroids, but achieved significant improvement after treatment with BTK inhibitors. These cases show the remarkable effectiveness of BTK inhibitors in refractory type II cryoglobulinemia without MyD88 mutation. To our knowledge, the successful use of BTK inhibitors in type II cryoglobulinemia with wild-type MyD88 has not been previously reported.

Mixed cryoglobulinemia is a rare condition and is clinically significant in approximately 1 in 100,000 individuals, with the majority being HCV-associated (7). Treatment of HCV-associated cryoglobulinemia typically involves immunosuppressive therapy followed by a combination of antiviral therapy (8). For non-HCV mixed cryoglobulinemia, evidence-based treatment is challenging due to its rarity. The treatment of all mixed cryoglobulinemia depends on the individual patient’s clinical presentation, severity of manifestations, and comorbidities (8). Plasmapheresis, a procedure that removes circulating cryoglobulins from the blood, is a rational therapeutic option for patients with severe disease (MPGN, leg ulcers) or life-threatening events (8). Immunosuppressive therapy, typically involving the use of corticosteroids along with other immunosuppressive agents and anti-CD20 monoclonal antibody, has shown effectiveness in inducing remission and improving symptoms (9–12). Although the combination of rituximab plus steroids yields a high clinical response, approximately 5% to 11% of patients remain refractory to this regimen (11). Even among patients with an initial clinical response, one-third experience an early relapse within the first year after induction therapy (9). In our cases, although both patients experienced improvement after treatment with steroids plus rituximab, they relapsed shortly even before the recovery of B cells. Therefore, innovative therapeutic approaches are strongly needed.

BTK is a TEC kinase with a multifaceted role in B-cell biology and function. It has been identified as a therapeutic target in various hematological malignancies due to its involvement in the B cell receptor signaling pathway, which is associated with cell proliferation and survival. Currently, five BTK inhibitors—ibrutinib, zanubrutinib, orelabrutinib, tirabrutinib, and acalabrutinib—have demonstrated remarkable efficacy and have been approved for the treatment of different types of hematological cancers (5). Some have shown effectiveness in treating cryoglobulinemia, primarily type I, which is mainly associated with Waldenstrom macroglobulinemia. Although type II cryoglobulinemia can also be caused by lymphoproliferative disorders, only two reported cases have suggested that BTK inhibitors were effective in type II cryoglobulinemia, both of which involved patients with a mutation in the MyD88 gene (13, 14). Therefore, whether BTK inhibitors are effective in type II cryoglobulinemia without MyD88 mutation remains completely unknown. In our initial case, a rapid and complete remission of nephrotic syndrome occurred within a month of initiating ibrutinib treatment, along with a notable decrease in cryoglobulin levels and abnormal clonal cells. This suggests that BTK inhibitors might hold promise as an effective approach in non-infectious type II cryoglobulinemia without MyD88 mutation, potentially due to its capacity to inhibit abnormal monoclonal cell generation and immunoglobulin production.

In our second case, the patient presented with HBV-associated type II cryoglobulinemia and had undergone multiple treatments before initiating BTK inhibitors. Initially, her rash disappeared after treatment with steroids plus cyclophosphamide, but reappeared when steroids were tapered. She then received a regimen of plasma exchange followed by rituximab, but her rash recurred, and she developed signs of skin ulceration and fatigue six months later, even before the recovery of B cells. Surprisingly, she experienced rapid disappearance of the rash within three days and rapid wound healing within one week after receiving orelabrutinib, along with a reduction in C-reactive protein. However, there was no reduction in cryoglobulin levels during a 12-month follow-up. Her symptoms, including rash, fatigue, and arthralgia, recurred when she discontinued orelabrutinib for several days due to COVID-19 infection, but disappeared immediately upon resuming the medication. These findings suggest that the efficacy of orelabrutinib may not be linked to clonal cell clearance or inhibition of immunoglobulin production.

It has been reported that acalabrutinib and ibrutinib are capable of inhibiting monocyte, macrophage, and neutrophil activation, thereby reducing the levels of inflammatory cytokines and chemokines such as IL-6, TNFα, IL-1, IFNγ and MCP-1 in patients with severe COVID-19 (15). BTK^-/-^monocytes, macrophages, and neutrophils show defects in TLR^-^ and NLRP3^-^ induced NF-κB activation, as well as impaired production of inflammatory cytokines and chemokines (16). Therefore, we potentially attribute the pharmacological effect of BTK inhibitors to their anti-inflammatory properties. Similar results have been found in another case report by Haya Jamali et al., in which they found that Ibrutinib can be remarkably effective in refractory cryoglobulinemia due to Waldenstrom macroglobulinemia with mutated MyD88, which may be attributed not only to its anti-tumor effect but also to its anti-inflammatory properties (14).

One main limitation of our cases is that we did not quantify the cryoglobulins or perform a specific analysis of inflammatory markers during the patient’s disease course. Nevertheless, this is the first report of patients with type II mixed cryoglobulinemia and wild-type MyD88 being successfully treated with BTK inhibitors. This report provides important information on the treatment of refractory type II mixed cryoglobulinemia.

In conclusion, cryoglobulinemia is a rare clinical condition, and its treatment presents challenges. There is a lack of high-quality evidence, especially for the less common non-HCV mixed cryoglobulinemia. Current treatments are typically aimed at addressing the underlying disease in combination with immunosuppressive therapy. Our cases suggest that BTK inhibitors may represent a new treatment option for patients with type II cryoglobulinemia who test negative for the MyD88 mutation. However, further study of this novel therapeutic approach for this rare disease is necessary.

## Data availability statement

The original contributions presented in the study are included in the article/supplementary material. Further inquiries can be directed to the corresponding author.

## Ethics statement

The studies involving humans were approved by Huashan Hospital, Fudan University. The studies were conducted in accordance with the local legislation and institutional requirements. Written informed consent for participation in this study was provided by the participants’ legal guardians/next of kin. Written informed consent was obtained from the individual(s) for the publication of any potentially identifiable images or data included in this article.

## Author contributions

MX: Data curation, Visualization, Writing – original draft. YX: Data curation, Methodology, Validation, Writing – review & editing, Resources. LY: Data curation, Methodology, Validation, Writing – review & editing. DS: Resources, Writing – review & editing. RC: Writing – review & editing. SL: Methodology, Writing – review & editing. YL: Resources, Writing – review & editing. AL: Resources, Writing – review & editing. RL: Resources, Writing – review & editing. QW: Writing – review & editing. TD: Writing – review & editing. QX: Data curation, Funding acquisition, Investigation, Resources, Supervision, Writing – review & editing. C-MH: Funding acquisition, Supervision, Writing – review & editing.

## References

[1] Brouet JC Clauvel JP Danon F Klein M Seligmann M. Biologic and clinical significance of cryoglobulins. Am J Med. (1974) 57:775–88. doi: 10.1016/0002-9343(74)90852-3 4216269

[2] Ramos-Casals M Stone JH Cid MC Bosch X. The cryoglobulinaemias. Lancet. (2012) 379:348–60. doi: 10.1016/S0140-6736(11)60242-0 21868085

[3] Guerrera ML Tsakmaklis N Xu L Yang G Demos M Kofides A. *MYD88* mutated and wild-type Waldenström’s Macroglobulinemia: characterization of chromosome 6q gene losses and their mutual exclusivity with mutations in. CXCR4. Haematologica. (2018) 103:e408–11. doi: 10.3324/haematol.2018.190181 PMC6119142 29599202

[4] Buske C Jurczak W Salem JE Dimopoulos MA. Managing Waldenström’s macroglobulinemia with BTK inhibitors. Leukemia. (2023) 37:35–46. doi: 10.1038/s41375-022-01732-9 36402930 PMC9883164

[5] Mendes-Bastos P Brasileiro A Kolkhir P Frischbutter S Scheffel J Moñino-Romero S. Bruton’s tyrosine kinase inhibition—An emerging therapeutic strategy in immune-mediated dermatological conditions. Allergy. (2022) 77:2355–66. doi: 10.1111/all.15261 PMC9545595 35175630

[6] Alu A Lei H Han X Wei Y Wei X. BTK inhibitors in the treatment of hematological Malignancies and inflammatory diseases: mechanisms and clinical studies. J Hematol Oncol. (2022) 15:138. doi: 10.1186/s13045-022-01353-w 36183125 PMC9526392

[7] Sansonno D Carbone A De Re V Dammacco F. Hepatitis C virus infection, cryoglobulinaemia, and beyond. Rheumatology. (2006) 46:572–8. doi: 10.1093/rheumatology/kel425 17317717

[8] Muchtar E Magen H Gertz MA. How I treat cryoglobulinemia. Blood. (2017) 129:289–98. doi: 10.1182/blood-2016-09-719773 27799164

[9] Terrier B Marie I Launay D Lacraz A Belenotti P de Saint-Martin L. Predictors of early relapse in patients with non-infectious mixed cryoglobulinemia vasculitis: Results from the French nationwide CryoVas survey. Autoimmunity Reviews (2014) 13(6):630–4. doi: 10.1016/j.lpm.2013.02.100 24418300

[10] Sneller MC Hu Z Langford CA. A randomized controlled trial of rituximab following failure of antiviral therapy for hepatitis C virus-associated cryoglobulinemic vasculitis: Rituximab in HCV-Associated Cryoglobulinemic Vasculitis. Arthritis Rheumatism. (2012) 64:835–42. doi: 10.1002/art.34322 PMC3243106 22147444

[11] Terrier B Krastinova E Marie I Launay D Lacraz A Belenotti P. Management of noninfectious mixed cryoglobulinemia vasculitis: data from 242 cases included in the CryoVas survey. Blood. (2012) 119:5996–6004. doi: 10.1182/blood-2011-12-396028 22474249

[12] Pouchelon C. Management of nonviral mixed cryoglobulinemia vasculitis refractory to rituximab: Data from a European collaborative study and review of the literature. Autoimmun Rev. (2022) 21(4):103034. doi: 10.1016/j.autrev.2022.103034 34995764

[13] Barot SV Lee SS Patel BJ Valent JN. Ibrutinib is effective in refractory type II cryoglobulinemia. Clin Lymphoma Myeloma Leukemia. (2019) 19:e629–32. doi: 10.1016/j.clml.2019.07.442 31585822

[14] Jamali H Wu D Soma L Linenberger M Wener MH Silberstein L. Ibrutinib response in a patient with refractory mixed essential cryoglobulinemia. eJHaem. (2023) 4:499–500. doi: 10.1002/jha2.686 37206249 PMC10188461

[15] Zhu S Gokhale S Jung J Spirollari E Tsai J Arceo J. Multifaceted immunomodulatory effects of the BTK inhibitors ibrutinib and acalabrutinib on different immune cell subsets – beyond B lymphocytes. Front Cell Dev Biol. (2021) 9:727531. doi: 10.3389/fcell.2021.727531 34485307 PMC8414982

[16] Zhu S Jung J Victor E Arceo J Gokhale S Xie P. Clinical trials of the BTK inhibitors ibrutinib and acalabrutinib in human diseases beyond B cell malignancies. Front Oncol. (2021) 11:737943. doi: 10.3389/fonc.2021.737943 34778053 PMC8585514

